# The Value of State Laws

**Published:** 1898-01

**Authors:** 


### THE VALUE OF STATE LAWS.

WE are pleased to publish in this issue a retrospect of the laws of Pennsylvania relative to the practice of veterinary science, and to note the sure and steadily favorable results growing out of the same.

These figures and their significance have been prepared by a member of the present senior class of students of the Veterinary Department of the University of Pennsylvania, and, as a prospective practitioner of his native State, his deductions fully prove how valuable he considers these laws in the interest of future fully-equipped practitioners.

Many other States now overrun with non-graduates, quacks, and illiterate men, where no laws exist, and where no laws were desired that recognized any but graduates, will surely realize the wisdom of those who, in Pennsylvania, advocated the present laws that were destined to make this State a fruitful field for educated members of the profession in the future, which time has been already reached in less than ten years. The figures show a change of over twenty-five per cent. already in those registered under the existing practitioner clause, and when a more careful examination of the records is made there will be found a much larger percentage.

Veterinarians will do well to gain an entering-wedge of legislation in every State, for its worth will be added to with each passing year.

